# mSphere of Influence: Rethinking microbial identity through variability

**DOI:** 10.1128/msphere.00026-26

**Published:** 2026-03-09

**Authors:** Laura Veschetti

**Affiliations:** 1 Infections and Cystic Fibrosis Unit, Division of Immunology, Transplantation and Infectious Diseases, IRCCS San Raffaele Scientific Institute 9372, Milano, Italy; 2 Vita-Salute San Raffaele University, Milano, Italy; Virginia-Maryland College of Veterinary Medicine, Blacksburg, Virginia, USA

**Keywords:** microbial variability, microbial evolution, population genomics

## Abstract

Laura Veschetti works in the field of microbial genomics and adaptation. In this mSphere of Influence article, she reflects on how the work of Marvig et al. on within-host evolution of *Pseudomonas aeruginosa*, together with long-term evolution experiments by Lenski and colleagues, reshaped her understanding of microbial identity by framing variability as a defining biological feature rather than an exception. Drawing on concepts from population genomics, pangenomics, and regulatory heterogeneity, she discusses how focusing on evolutionary processes rather than static reference genomes has influenced her approach to studying bacterial adaptation.

## COMMENTARY

I remember when I first learned about DNA in high school: everything seemed to be governed by precise, tightly regulated mechanisms and unbreakable laws. All processes unfolded like clockwork, and cell life felt very logical: in order to exist, life needs to be algorithmic. Changes to the ingredients or to the instructions are deleterious, if not fatal. This view began to change as I learned about experimental and natural evolution in bacteria. A first turning point was the *Escherichia coli* long-term evolution experiment initiated by Richard Lenski in 1988 (1), in which 12 initially identical populations evolved independently for over 75,000 generations, accumulating extensive phenotypic and genetic diversity. This work raised a deceptively simple question that has stayed with me since: when do we stop being the same organism? This question resurfaced during my PhD when I read the study by Marvig and colleagues on the evolution and adaptation of *Pseudomonas aeruginosa* within patients with cystic fibrosis (2). By showing that distinct lineages of the same species undergo parallel yet highly diverse evolutionary trajectories within individual hosts, this work fundamentally reshaped how I think about microbial identity and species boundaries in bacteria. It became clear that variation is not the exception, but rather the rule. DNA accumulates mutations, organisms evolve, and controlled chaos and creativity are necessary. Taxonomy implies that there are strict criteria to be given a name and clear limits that define each organism from all other forms of life. Nonetheless, in both cases, what started as a genomically and phenotypically homogeneous population became a rich and complex mosaic. Hypermutation, horizontal transfer, gene expression, protein production, and localization are all key mechanisms that introduce variability: the one true feature that allows us to be life in relation to the environment. The focus of (micro)biology is not on static organisms, but on processes.

The impact of these works on my thinking did not stem from their analytical framework but from the striking mismatch between the richness of the evolutionary trajectories observed and the tools used to describe them. Reading this paper forced me to reconsider how we typically study genomic adaptation: the standard practice is to identify the bacteria and compare their sequence to that of a reference genome for that species. Genomically speaking, belonging to the same species means that the two sequences display a high average nucleotide identity. This means that the criterion for belonging to the same taxon is a similarity threshold calculated on shared protein-coding genes. What about the rest of the genomic content? Information related to additional genes or mobile genetic elements is disregarded; they are just the “little variation” between the highly virulent *E. coli* O157:H7 and the commensal *E. coli* K-12 genomes. Moreover, we are not only comparing two sequences but also two consensus sequences resulting from the combination of the most frequent nucleotide in that bacterial population at each genomic position. In other words, this is like describing a city by averaging all its inhabitants into a single “mean person”: the result is neat and convenient, but it erases individuality and complexity. If brought to extremes, this could mean that we are comparing two sequences that might not even exist. In this context, it could be worth exploring the possibility of using population genomics approaches to study bacterial adaptation. This would allow us to capture and better compare bacterial genomic richness and variability.

Similar insights emerge when shifting perspective from individual genomes to species-wide pangenomes. Once again, bacterial genomic richness and plasticity become evident: the core genome of many species comprises only roughly half of the average gene content for that species, with some taxa (e.g., *Achromobacter xylosoxidans* and *Klebsiella michiganensis*) comprising only 30% of the average gene content. “A rose by any other name may smell as sweet,” but we call widely different bacteria by the same name. Should we change the point of view to that of transcriptomics, proteomics, metabolomics, or phenotypic and biochemical assays, the picture becomes even more diverse and multifaceted. In their work, both Lenski and Marvig witnessed the emergence of novel phenotypes through a plethora of adaptation strategies that go beyond genomic adaptation. A first, quicker level of adaptive response is obtained through transcript regulation. Over the last decade, research has shed light on the existence of small regulatory RNAs that play a key role in regulating bacterial virulence and phenotypes. In this context, variability is introduced into the system not only by the differential expression level but also by the very limited conservation of these molecules across different species and conditions (3). Indeed, the interaction between organisms and the environment significantly contributes to variability. For example, we know that bacteria can display different phenotypes under *in vivo* and *in vitro* conditions, and this is also due to population dynamics (4). Technological advancements now enable us to achieve single-cell resolution, but we need tools and frameworks that allow us to collect and synthesize variability at the population level.

Bacteria taught me that identity is not something you have, but something you negotiate with your environment. Like Pirandello’s characters, bacteria are never just one. They are shaped by context, perceived through function, and defined by interaction: one, no one, and one hundred thousand.
